# Author Correction: Resistance to CDK7 inhibitors directed by acquired mutation of a conserved residue in cancer cells

**DOI:** 10.1038/s44318-026-00862-5

**Published:** 2026-07-17

**Authors:** Chun-Fui Lai, Victoria I Cushing, Ellen Olden, Charlotte L Bevan, R Charles Coombes, Basil J Greber, Laki Buluwela, Simak Ali

**Affiliations:** 1 Department of Surgery & Cancer, Imperial, London, UK; 2 Institute of Cancer Research, Chester Beatty Laboratories, London, UK

## Abstract

**Figure:**
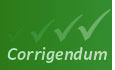

**Correction to:** *The EMBO Journal* (2025) 44: 5860–5889. 10.1038/s44318-025-00554-6 | Published online 08 September 2025

The authors contacted the journal to inform them that there is an error in the Acknowledgements section.

**The Acknowledgements section is corrected**.

The Acknowledgements section is corrected from:

We thank S. Omarjee, J. Carroll and A. Bahl for their generous gift of cell lines and samuraciclib, respectively. We thank C. Richardson for support with high-performance computing, R. Knight for support of insect cell culture, and K. Kondas, M. Stubbs, S. Hearnshaw, and R. van Montfort for help with biophysical assays. SA, CLB, LB and C-FL received funding for this work from Prostate Cancer UK (grant number RIA19-ST2-00). BJG was supported by a career development fellowship from the Medical Research Council of the UK (grant number MR/V009354/1). VIC was funded by an ICR PhD studentship. EO was funded by the Imperial MRC MultiSci doctoral training partnership PhD program, with industrial funding from Carrick Therapeutics.

To: **Changes in bold**.

We thank S. Omarjee, J. Carroll and A. Bahl for their generous gift of cell lines and samuraciclib, respectively. We thank C. Richardson for support with high-performance computing, R. Knight for support of insect cell culture, and K. Kondas, M. Stubbs, S. Hearnshaw, and R. van Montfort for help with biophysical assays. SA, CLB, LB and C-FL received funding for this work from Prostate Cancer UK (grant number **RIA19-ST2-004**). BJG was supported by a career development fellowship from the Medical Research Council of the UK (grant number MR/V009354/1). VIC was funded by an ICR PhD studentship. EO was funded by the Imperial MRC MultiSci doctoral training partnership PhD program, with industrial funding from Carrick Therapeutics.

