# Supplementary material for: Resistance to CDK7 inhibitors directed by acquired mutation of a conserved residue in cancer cells
Source: EMBO J. 2025 Sep 8;44(20):5860–89. doi: 10.1038/s44318-025-00554-6 (PMC12528448; doi:10.1038/s44318-025-00554-6)
Supplement: Supplementary file 1 — Table EV1 [file 44318_2025_554_MOESM1_ESM.docx]

**Table EV1. Statistics for cryo-EM data collection, 3D reconstruction, model refinement, and validation.**

| **Ligand** | **ATPγS** | **Samuraciclib** | **THZ1** |
| --- | --- | --- | --- |
| Microscope | Krios | Krios | Krios |
| Stage type | Autoloader | Autoloader | Autoloader |
| Voltage (kV) | 300 | 300 | 300 |
| Detector | K3 | K3 | Falcon 4i |
| Energy filter | BioQuantum | BioQuantum | Selectris X |
| Acquisition mode | Counting | Counting | Counting |
| Pixel size (Å) | 0.51 | 0.513 | 0.576 |
| Defocus range (μm) | 0.7-2.2 | 0.6-2.0 | 0.7-1.9 |
| Electron exposure (e^-^/A^2^) | 63 | 70 | 70 |
| **Reconstruction** | **EMD-52206** | **EMD-52207** | **EMD-52205** |
| Software | RELION 5.0 | RELION 5.0 | RELION 5.0 |
| Particles used | 364,199 | 133,495 | 956,820 |
| Box size (pixels) | 160 x 160 x 160 | 180 x 180 x 180 | 180 x 180 x 180 |
| Final pixel size (Å) | 1.02 | 1.026 | 1.152 |
| Accuracy rotations (°) | 1.44 | 1.11 | 1.20 |
| Accuracy translations (Å) | 0.44 | 0.36 | 0.37 |
| Map resolution (Å) | 2.3 | 2.6 | 2.6 |
| Map resolution range | 2.2-3.0 | 2.5-3.2 | 2.5-2.9 |
| Sphericity | 0.92 | 0.87 | 0.95 |
| Map sharpening B-factor (Å^2^) | -50 | -56 | -87 |
| **Coordinate refinement** |  |  |  |
| Software | PHENIX | PHENIX | PHENIX |
| Refinement algorithm | REAL SPACE | REAL SPACE | REAL SPACE |
| Resolution cutoff (Å) | 2.3 | 2.6 | 2.6 |
| FSC_model-vs-map_=0.5 (Å) | 2.4 | 2.7 | 2.6 |
| **Model** | **PDB-9HIY** | **PDB-9HJ0** | **PDB-9HIX** |
| Number of residues | 651 | 659 | 656 |
| Protein | 624 | 618 | 625 |
| Ligand (inhibitor, Mg^2+^) | 3 | 2 | 2 |
| Water | 24 | 39 | 59 |
| B-factors overall | 46.12 | 37.22 | 27.65 |
| Protein | 46.08 | 37.39 | 27.61 |
| Ligand (inhibitor, Mg^2+^) | 62.59 | 31.17 | 37.81 |
| Water | 29.11 | 24.33 | 16.46 |
| R.M.S. deviations |  |  |  |
| Bond lengths (Å) | 0.002 | 0.003 | 0.002 |
| Bond angles (°) | 0.547 | 0.533 | 0.489 |
| **Validation** |  |  |  |
| Molprobity score | 1.38 | 1.52 | 1.24 |
| Molprobity clashscore | 5.61 | 6.49 | 4.71 |
| Rotamer outliers (%) | 1.27 | 1.65 | 0.91 |
| C_β_ deviations (%) | 0.00 | 0.00 | 0.00 |
| Ramachandran plot |  |  |  |
| Favored (%) | 98.68 | 98.01 | 98.69 |
| Allowed (%) | 1.15 | 1.83 | 1.15 |
| Outliers (%) | 0.16 | 0.17 | 0.16 |
