# Supplementary figures and images for: Resistance to CDK7 inhibitors directed by acquired mutation of a conserved residue in cancer cells
Source: EMBO J. 2025 Sep 8;44(20):5860–89. doi: 10.1038/s44318-025-00554-6 (PMC12528448; doi:10.1038/s44318-025-00554-6)

## Slide 1
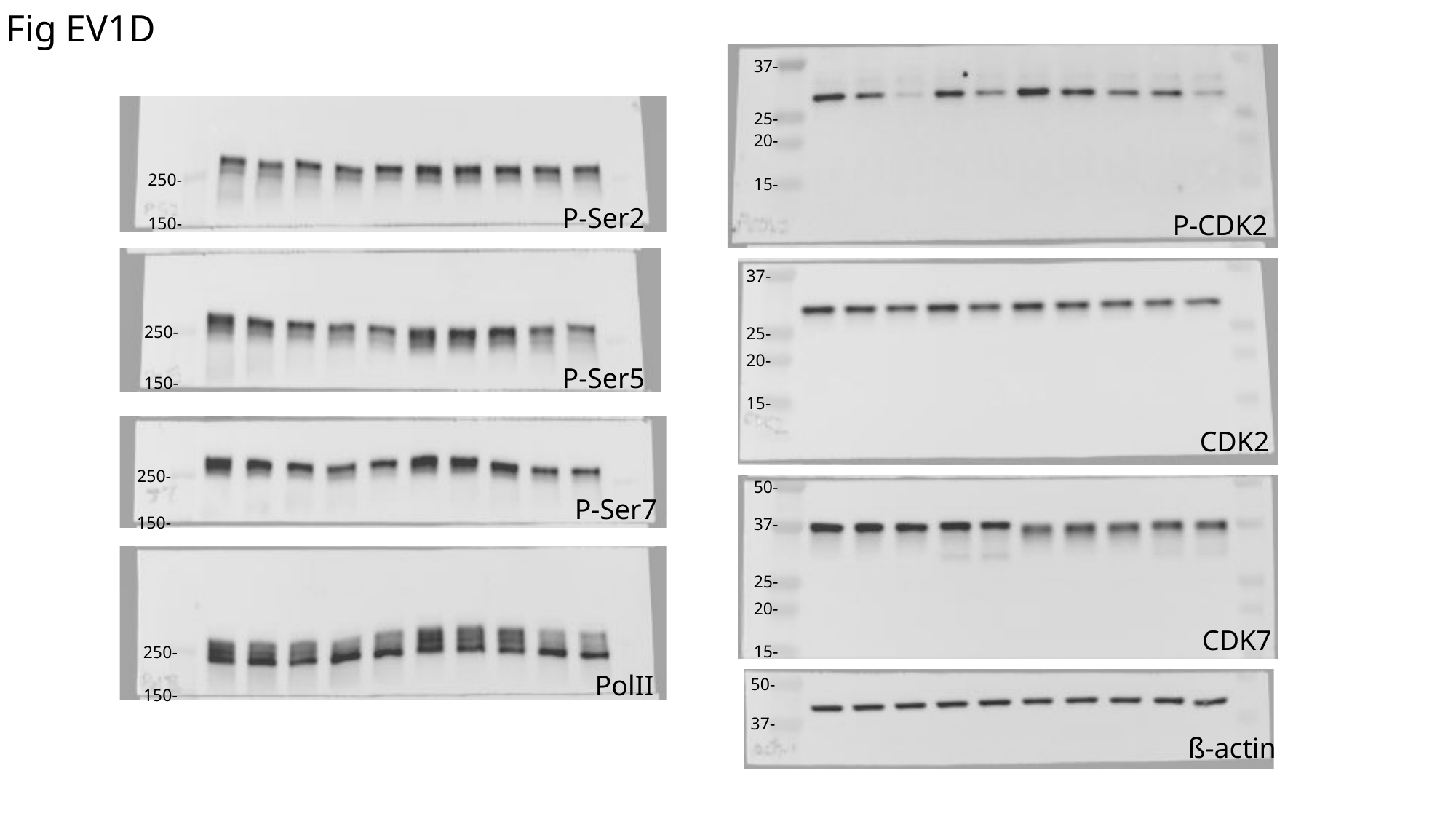

Fig EV1D
37-
25-
20-
250-
15-
P-Ser2
P-CDK2
150-
37-
250-
25-
20-
P-Ser5
150-
15-
CDK2
250-
50-
P-Ser7
150-
37-
25-
20-
CDK7
15-
250-
PolII
50-
150-
37-
ß-actin

Supplement: Supplementary file 3 — Source data Fig. 1 [file 44318_2025_554_MOESM3_ESM.zip › Fig EV1 image source data.pptx]

## Slide 1
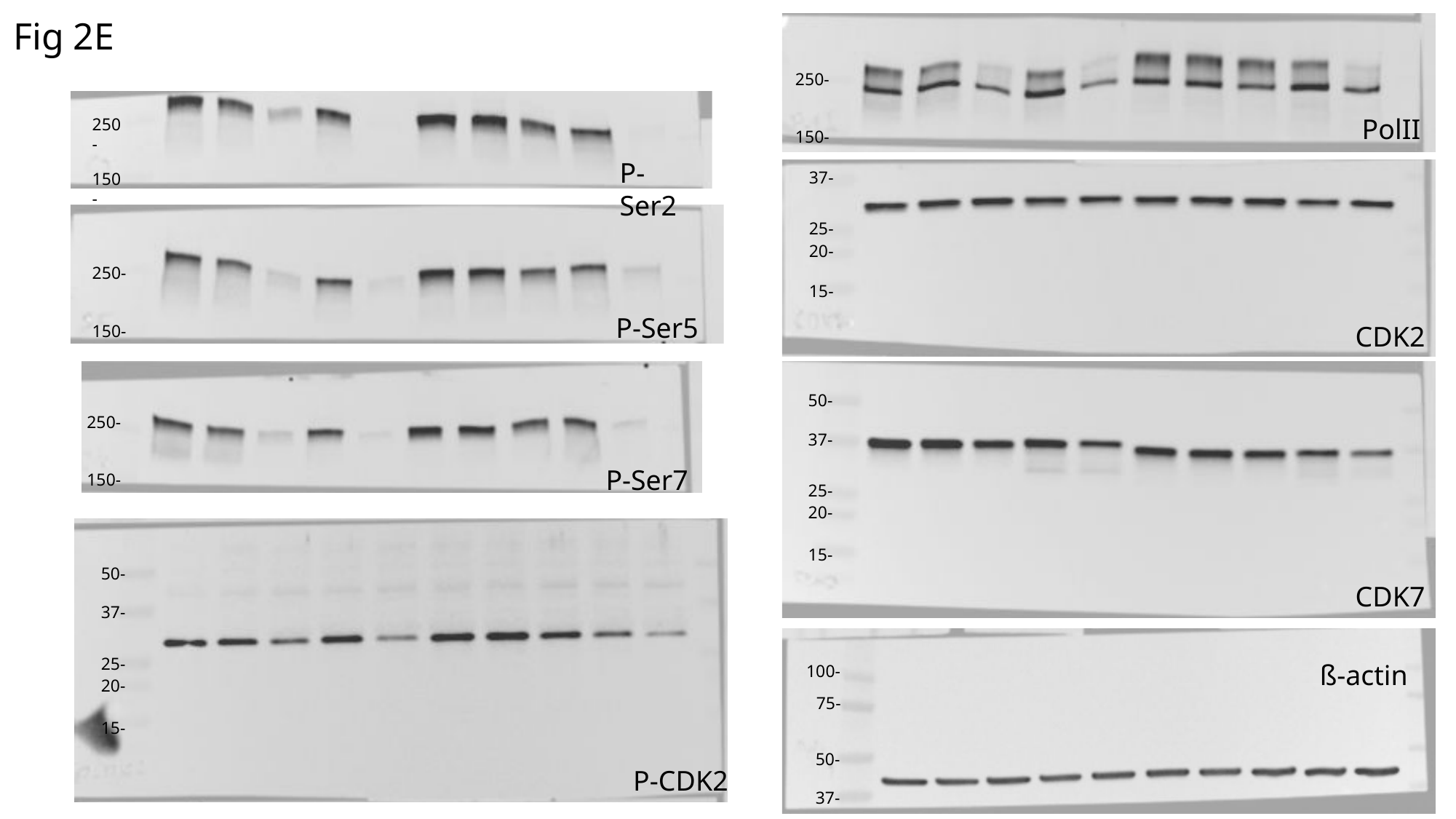

Fig 2E
250-
PolII
250-
150-
P-Ser2
37-
150-
25-
20-
250-
15-
P-Ser5
CDK2
150-
50-
250-
37-
P-Ser7
150-
25-
20-
15-
50-
CDK7
37-
25-
ß-actin
100-
20-
75-
15-
50-
P-CDK2
37-

Supplement: Supplementary file 4 — Source data Fig. 2 [file 44318_2025_554_MOESM4_ESM.zip › Figure 2E source data.pptx]

## Slide 1
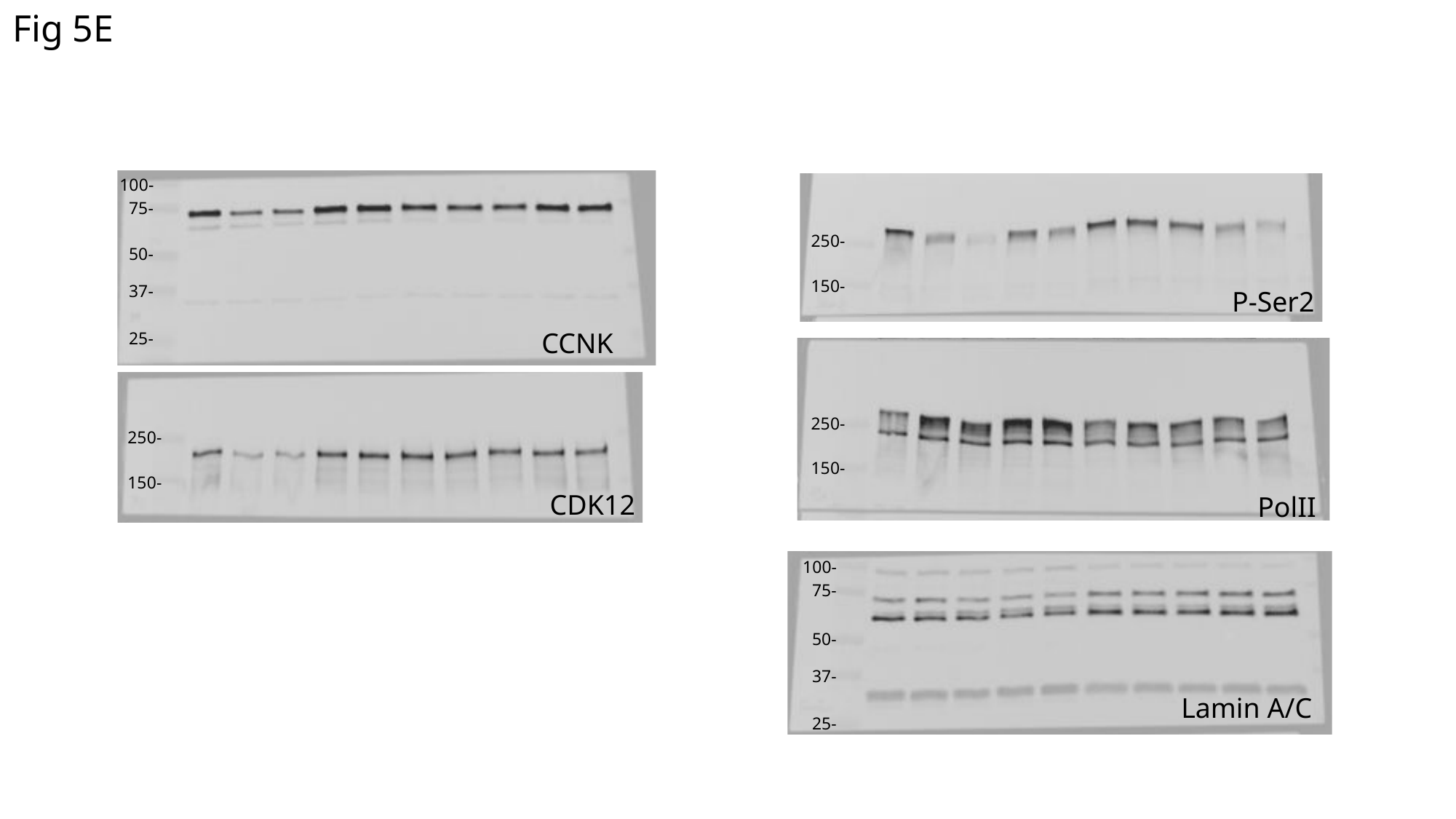

Fig 5E
100-
75-
250-
50-
150-
37-
P-Ser2
CCNK
CCNK
25-
250-
250-
150-
150-
CDK12
PolII
100-
75-
50-
37-
Lamin A/C
25-

Supplement: Supplementary file 6 — Source data Fig. 5 [file 44318_2025_554_MOESM6_ESM.zip › Figure 5E source data images.pptx]
