# Supplementary material for: Resistance to CDK7 inhibitors directed by acquired mutation of a conserved residue in cancer cells
Source: EMBO J. 2025 Sep 8;44(20):5860–89. doi: 10.1038/s44318-025-00554-6 (PMC12528448; doi:10.1038/s44318-025-00554-6)
Supplement: Supplementary file 7 — Source data Fig. 6 [file 44318_2025_554_MOESM7_ESM.zip › Figure 6C Image source data.pptx]

## Slide 1
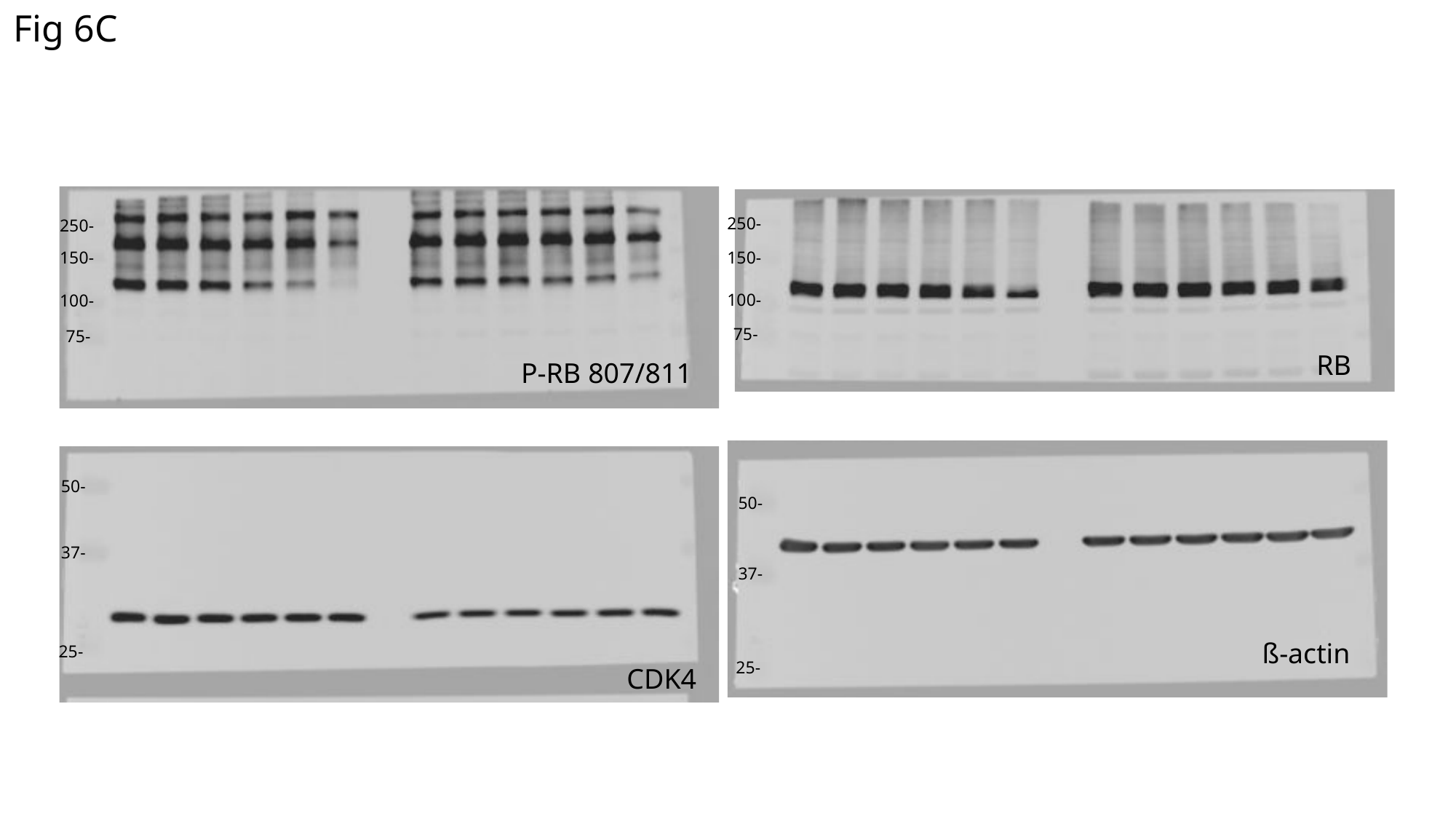

Fig 6C
250-
250-
150-
150-
100-
100-
75-
75-
RB
P-RB 807/811
50-
50-
37-
37-
ß-actin
25-
25-
CDK4

## Slide 2
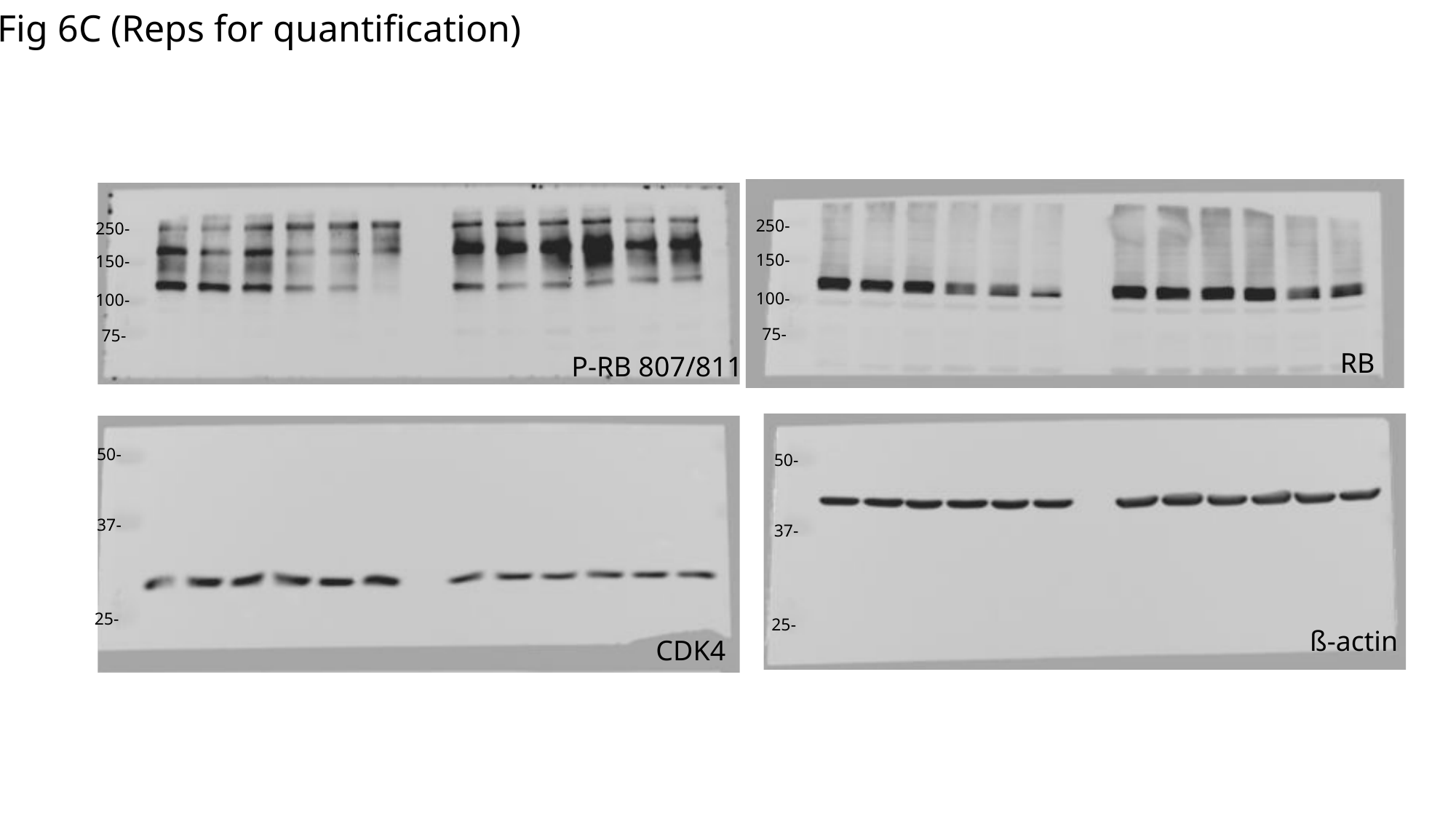

Fig 6C (Reps for quantification)
250-
250-
150-
150-
100-
100-
75-
75-
RB
P-RB 807/811
50-
50-
37-
37-
25-
25-
ß-actin
CDK4

## Slide 3
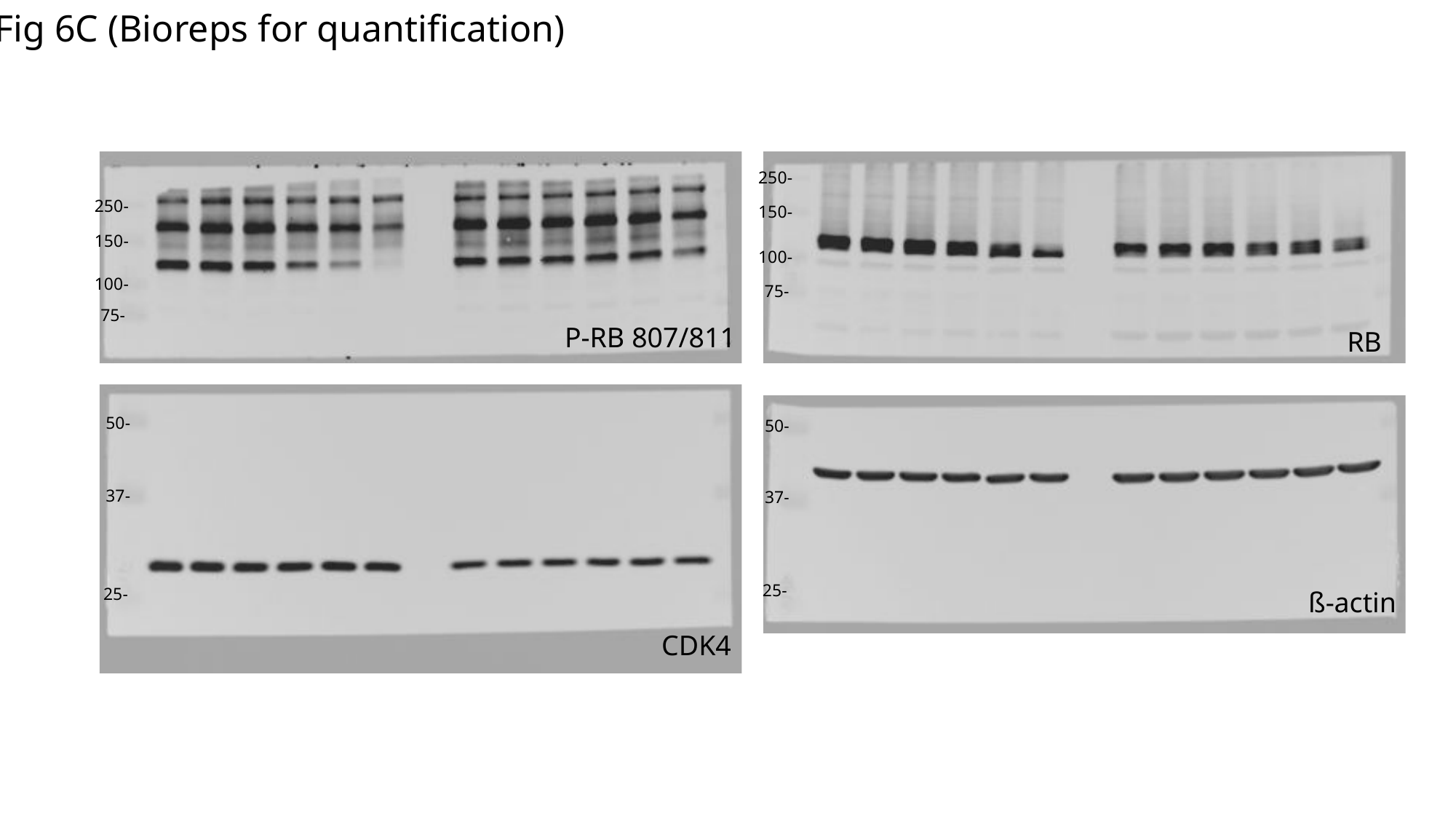

Fig 6C (Bioreps for quantification)
250-
250-
150-
150-
100-
100-
75-
75-
P-RB 807/811
RB
50-
50-
37-
37-
25-
25-
ß-actin
CDK4
