# Supplementary material for: Resistance to CDK7 inhibitors directed by acquired mutation of a conserved residue in cancer cells
Source: EMBO J. 2025 Sep 8;44(20):5860–89. doi: 10.1038/s44318-025-00554-6 (PMC12528448; doi:10.1038/s44318-025-00554-6)
Supplement: Supplementary file 10 — Figure EV3 Source Data [file 44318_2025_554_MOESM10_ESM.zip › Image source data_SA EVs.pptx]

## Slide 1
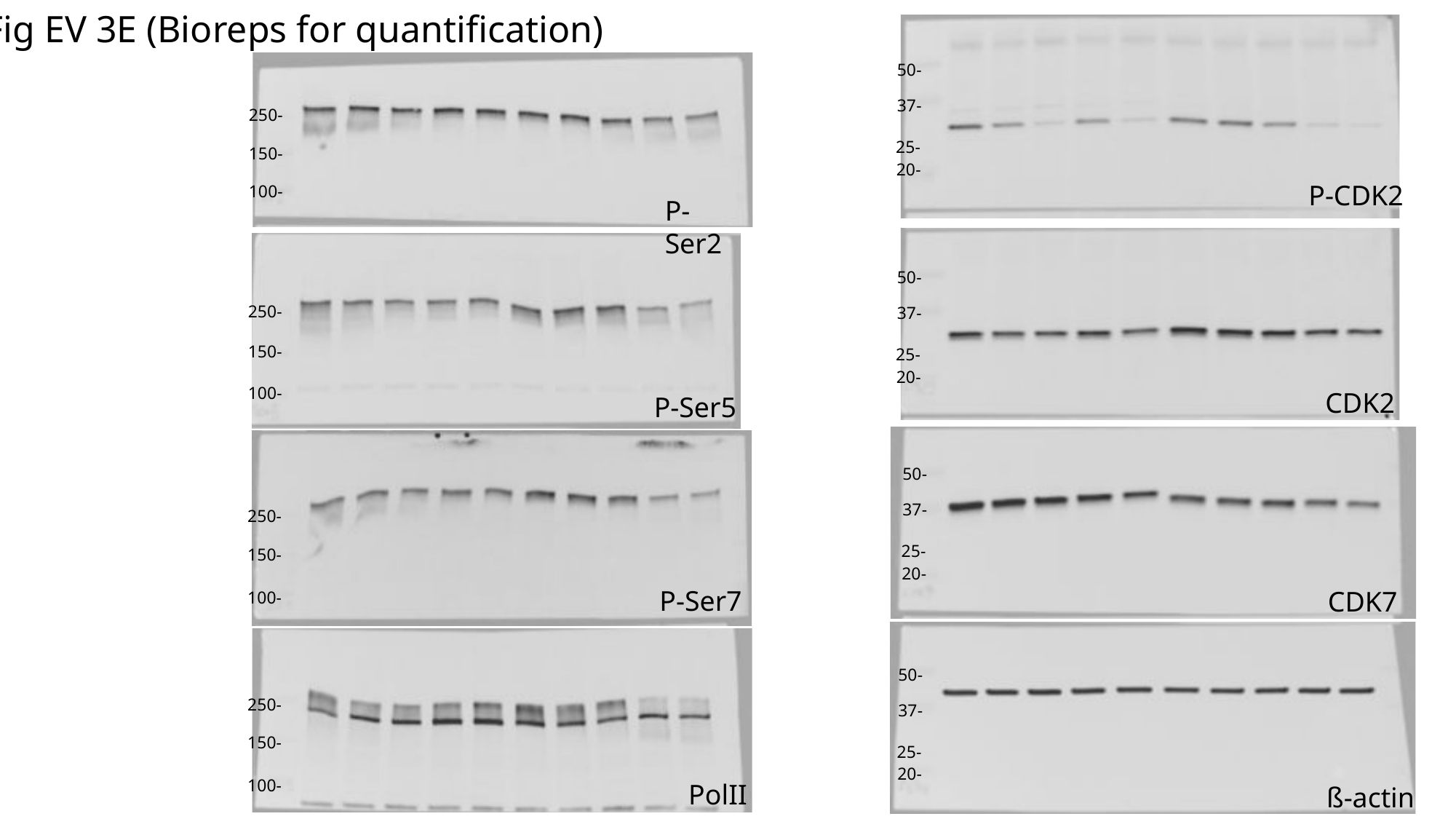

Fig EV 3E (Bioreps for quantification)
50-
37-
250-
25-
150-
20-
P-CDK2
100-
P-Ser2
50-
250-
37-
150-
25-
20-
100-
CDK2
P-Ser5
50-
37-
250-
25-
150-
20-
P-Ser7
CDK7
100-
50-
250-
37-
150-
25-
20-
100-
PolII
ß-actin

## Slide 2
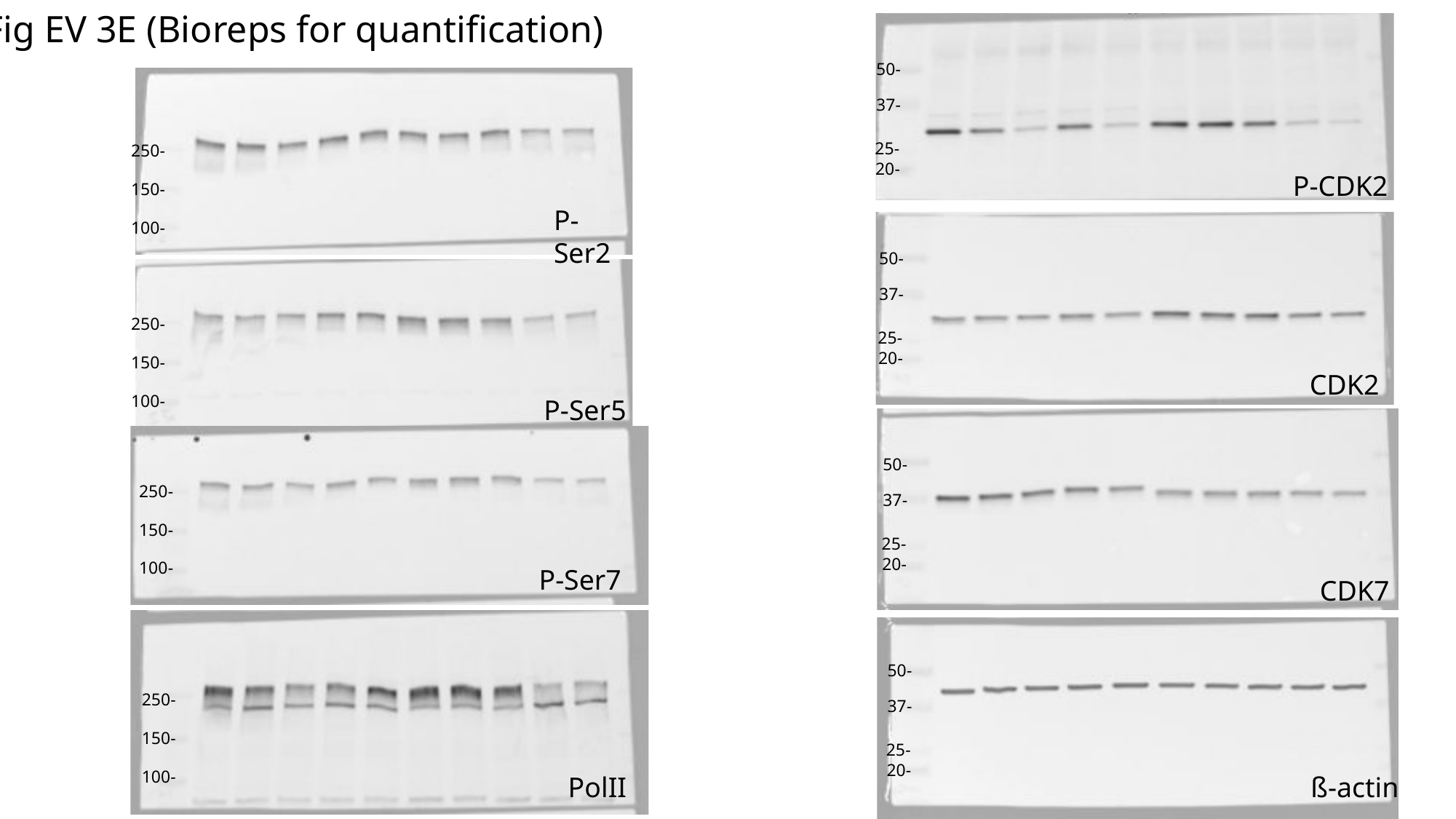

Fig EV 3E (Bioreps for quantification)
50-
37-
25-
250-
20-
P-CDK2
150-
P-Ser2
100-
50-
37-
250-
25-
20-
150-
CDK2
100-
P-Ser5
50-
250-
37-
150-
25-
20-
100-
P-Ser7
CDK7
50-
250-
37-
150-
25-
20-
100-
PolII
ß-actin
