# Supplementary material for: Resistance to CDK7 inhibitors directed by acquired mutation of a conserved residue in cancer cells
Source: EMBO J. 2025 Sep 8;44(20):5860–89. doi: 10.1038/s44318-025-00554-6 (PMC12528448; doi:10.1038/s44318-025-00554-6)
Supplement: Supplementary file 12 — Expanded View Figures [file 44318_2025_554_MOESM12_ESM.pdf]

Expanded View Figures

Figure EV1. Samuraciclib-resistant 22Rv1 cells are cross-resistant to other ATP-competitive CDK7 inhibitors.

(**A**) Mean IC_{50} values from $n = 3$ independent experiments for 22Rv1 and 22Rv1-SamR cells. These results are summarised in Fig. 1. Error bars = SEM. (**B**) Densitometric quantification of immunoblots from $n = 3$ independent experiments in which 22Rv1 and 22Rv1-SamR cells were treated with the indicated concentrations of Samuraciclib or SY1365. Signal intensities are shown relative to the appropriate vehicle (0 nM) controls. One of the immunoblots used for the quantification is shown in Fig. 1D. Error bars show SEM. Pairwise comparisons between treated groups and vehicle were performed using repeated measures of one-way ANOVA followed by Fisher's LSD post hoc test (uncorrected). Asterisks indicate significance (*$P < 0.05$, **$P < 0.01$). P value for P-Ser5, 22Rv1 cells: Veh vs Sam (100 nM) $P = 0.0093$; Veh vs Sam (1000 nM) $P = 0.0232$. 22Rv1-SamR cells: Veh vs SY1365 (5 nM) $P = 0.0227$; Veh vs SY1365 (50 nM) $P = 0.0052$. For P-CDK2, 22Rv1 cells: Veh vs Sam (100 nM) $P = 0.0148$; Veh vs Sam (1000 nM) $P = 0.0017$; Veh vs SY1365 (50 nM) $P = 0.0268$. 22Rv1-SamR cells: Veh vs Sam (1000 nM) $P = 0.0027$; Veh vs SY1365 (50 nM) $P = 0.0102$. For β-actin, 22Rv1-SamR cells: Veh vs sam (1000 nM) $P = 0.0441$. No asterisk indicates a non-significant difference ($P > 0.05$). (**C**) RNA-seq was performed using six RNA samples prepared from 22Rv1 and 22Rv1-SamR cells. Shown are normalised read counts for CDK7 and its interacting partners cyclin H (CCNH) and MAT1 (MNAT1). Circles represent the normalised read counts for each of the six biological replicate RNA samples. (**D**, **E**) Genome browser snapshots (hg38) of RNA-seq data for 22Rv1 (SAMSEN) and 22Rv1-SamR (SAMRES) cells are shown. The vertical bars show positions of two known CDK7 SNPs (rs2972388 (exon 2) and rs34584424 (exon 10)), which are present in the Sam-sensitive and resistant cells at a ratio of 1:1, suggestive of the presence of 2 CDK7 alleles in 22Rv1 cells. Also evident is a single nucleotide difference (exon 5; c.289G>A) in the codon encoding aspartate 97 (p.Asp97Asn), which is seen only in 22Rv1-SamR cells. Source data are available online for this figure.

 © The Author(s)

A

		IC50 (nM)		95% CI	
		22Rv1	22Rv1-Sam	22Rv1	22Rv1-Sam
**Non-covalent**	Samuraciclib	107.1	1719	93.3 to 123.1	1473 to 2005
	LDC4297	38.9	1717	32.9 to 45.0	1503 to 1962
	SY-5609	3.7	5136	3.0 to 4.7	4326 to 6124
**Covalent**	SY-1365	2.4	2.4	2.1 to 2.7	1.8 to 3.3
	THZ1	21.4	13.9	19.4 to 23.9	12.2 to 15.8
	YKL-5-124	6.2	12.4	5.4 to 7.1	10.5 to 14.6

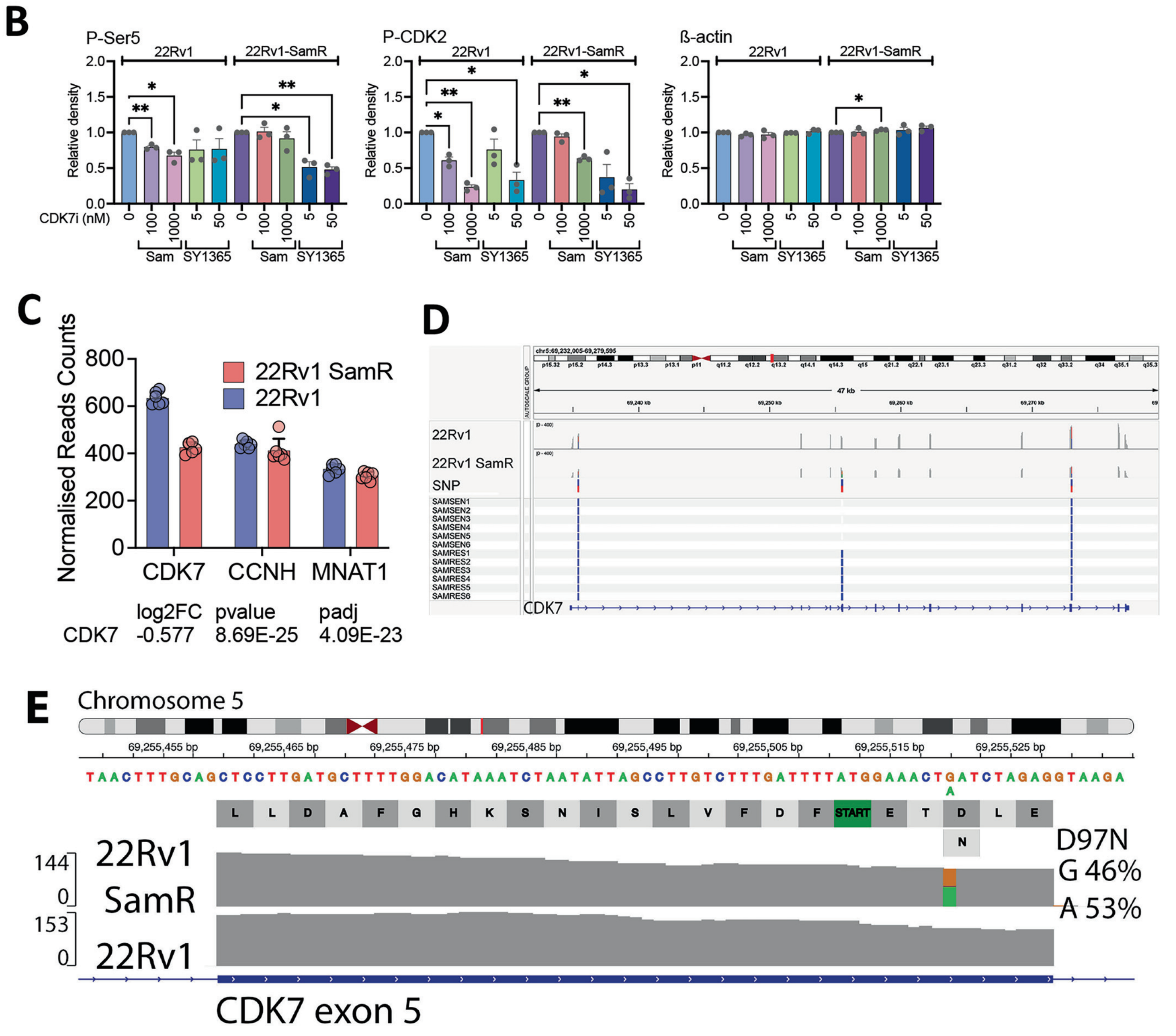

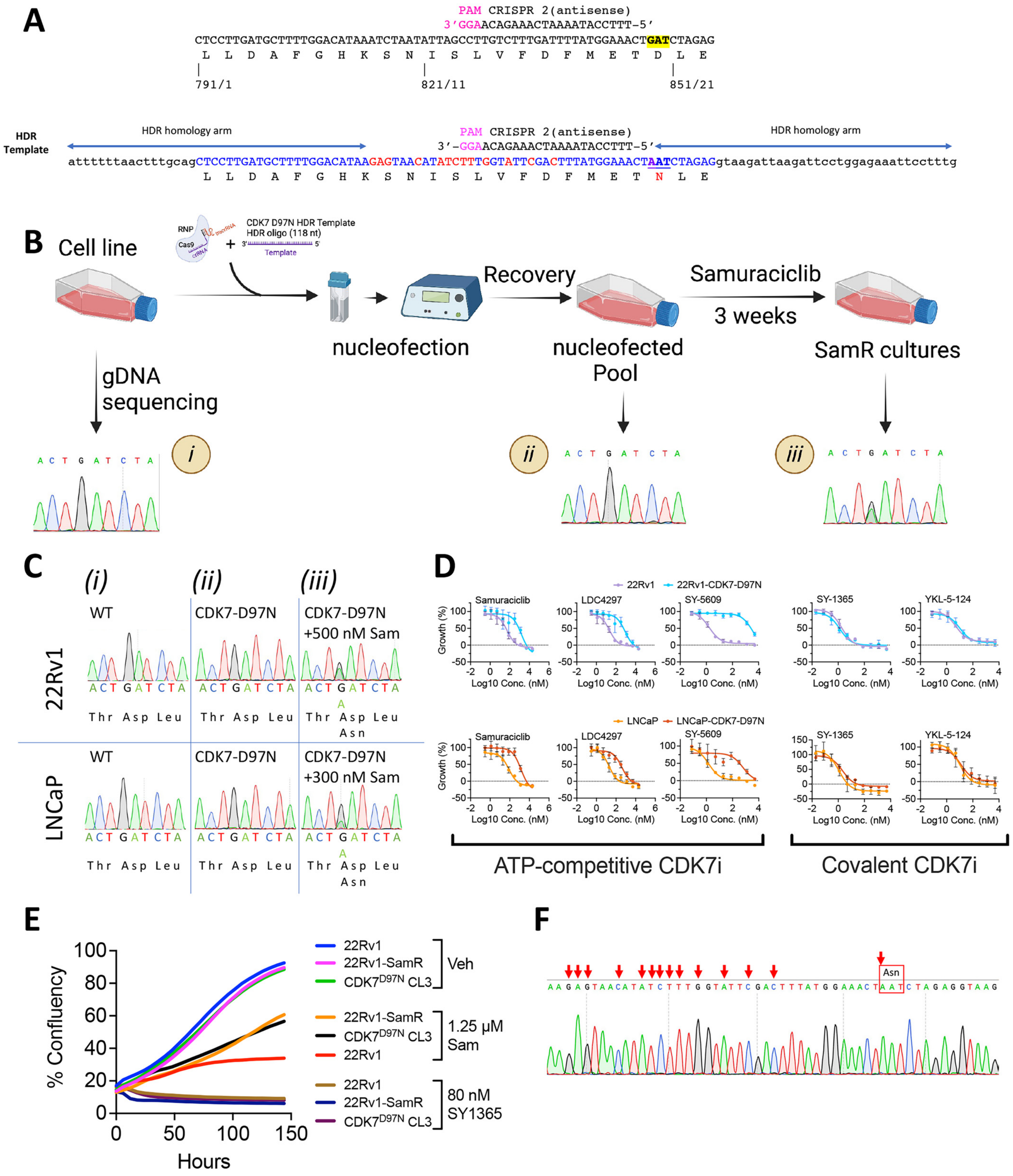
A
PAM CRISPR 2(antisense)
3′GGAACAGAAACTAAAATACCTTT-5′
CTCCTTGATGCTTTTGGACATAAATCTAATATTAGCCTTGTCTTTGATTTTATGGAAACTGATCTAGAG
L L D A F G H K S N I S L V F D F M E T D L E
791/1
821/11
851/21
HDR Template
HDR homology arm
PAM CRISPR 2(antisense)
3′-GGAACAGAAACTAAAATACCTTT-5′
HDR homology arm
atttttaactttgcagCTCCTTGATGCTTTTGGACATAAGAGTAACATATCTTTGGTATTCGACTTTATGGAAACTAATCTAGAGgtaagattaagattcctggagaaattcctttg
L L D A F G H K S N I S L V F D F M E T N L E
B
Cell line
RNP
Cas9
CDK7 D97N HDR Template
HDR oligo (118 nt)
Template
nucleofection
Recovery
nucleofected Pool
Samuraciclib
3 weeks
SamR cultures
gDNA sequencing
i
ii
iii
C
(i)
(ii)
(iii)
22Rv1
WT
CDK7-D97N
CDK7-D97N +500 nM Sam
Thr Asp Leu
Asn
LNCaP
WT
CDK7-D97N
CDK7-D97N +300 nM Sam
Thr Asp Leu
Asn
D
22Rv1
22Rv1-CDK7-D97N
Samuraciclib
LDC4297
SY-5609
SY-1365
YKL-5-124
Growth (%)
Log10 Conc. (nM)
LNCaP
LNCaP-CDK7-D97N
ATP-competitive CDK7i
Covalent CDK7i
E
% Confluency
Hours
22Rv1
22Rv1-SamR
CDK7D97N CL3
Veh
1.25 µM Sam
80 nM SY1365
F
Asn

Figure EV2. Development of CDK7-D97N mutation in cancer cell lines using CRISPR/Cas9 mutagenesis.

(**A**, **B**) Shown are the CRISPR sequences and the sequence of the homology-directed recombination (HDR) template for introduction of the CDK7-D97N mutation. The sequences in blue show the CDK7 exon 5 sequences, and the sequences in red represent silent changes introduced in the template for facilitating screening for the mutation. Underlined is the codon encoding asparagine at position 97 to create the p.Asp97Asn (D97N) mutation in the CDK7 gene. (**B**) The strategy for enrichment of cells encoding CDK7-D97N. (**C**) Sequencing chromatograms for the region around Asp97 in unmutated cells (i) in gDNA prepared from 22Rv1 and LNCaP cells. Sequencing chromatograms for cells following nucleofection and culturing in the absence (ii) or the presence (iii) of Sam. (**D**) Growth inhibition for increasing concentrations of the indicated drugs was carried out using Sam-selected D97N knock-in cells (22Rv1-CDK7-D97N, LNCaP-CDK7-D97N (iii)) alongside parental Sam-sensitive cells; means and SEM from $n = 2$ independent experiments are shown. (**E**) The cell lines were cultured in the presence of the indicated drugs, with confluency being measured every 6 h using an Incucyte Zoom. Non-linear regression analysis was used for curve fitting. The results of $n = 3$ independent experiments are summarised, with error bars omitted for ease of viewing. Calculated doubling times with 95% confidence intervals (h) for vehicle-treated cells: 22Rv1 (49.4; 45.3–54.1), 22Rv1-SamR (45.4; 42.2–49.0), 22Rv1 homozygous CDK7-D97N CL3 (48.8; 45.1–53.0). (**F**) Sanger sequencing chromatogram for gDNA prepared from the 22Rv1 CDK7-D97N CL3. Source data are available online for this figure.

© The Author(s)

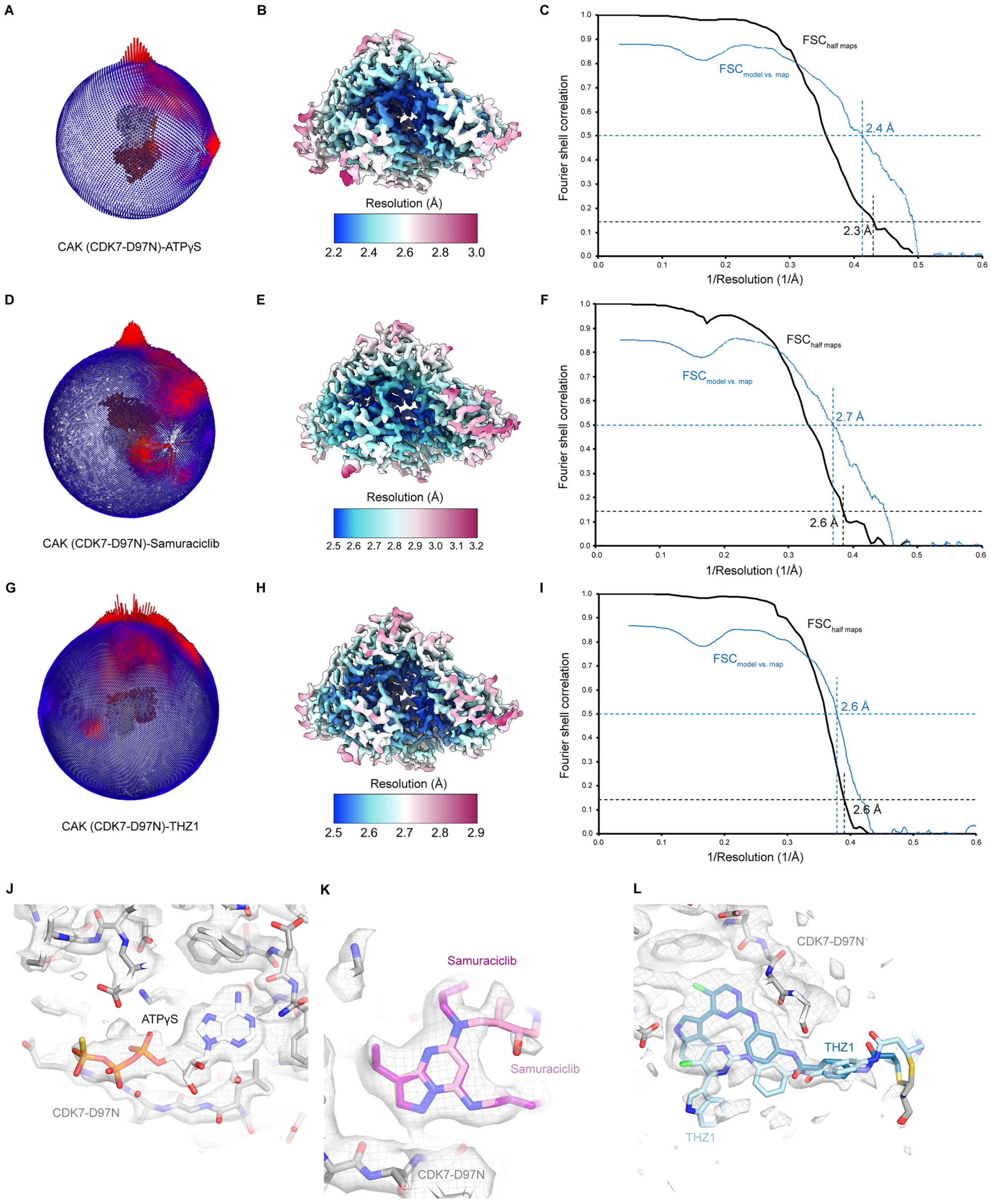
A
B
C
CAK (CDK7-D97N)-ATPγS
Resolution (Å)
2.2 2.4 2.6 2.8 3.0
FSC half maps
FSC model vs. map
2.4 Å
2.3 Å
Fourier shell correlation
1/Resolution (1/Å)
D
E
F
CAK (CDK7-D97N)-Samuraciclib
2.5 2.6 2.7 2.8 2.9 3.0 3.1 3.2
2.7 Å
2.6 Å
G
H
I
CAK (CDK7-D97N)-THZ1
2.5 2.6 2.7 2.8 2.9
2.6 Å
J
K
L
ATPγS
CDK7-D97N
Samuraciclib
THZ1

 © The Author(s)

Figure EV3. Validation measures for the cryo-EM reconstructions and additional ligand views.

(**A**) Plot of the orientation distribution of the particle images entering the CAK D97N-ATPγS cryo-EM reconstruction. (**B**) Local resolution estimation for the CAK D97N-ATPγS cryo-EM reconstruction. (**C**) FSC plots to assess the overall resolution of the CAK D97N-ATPγS cryo-EM reconstruction (black line) and the model-to-map fit of the refined coordinate model (blue line). FSC thresholds of FSC = 0.143 and FSC = 0.5 were used for half-map and model-to-map comparisons, respectively (Rosenthal and Henderson, 2003). (**D–F**) As (**A–C**), but for the CAK D97N-Samuraciclib cryo-EM reconstruction. (**G–I**) As (**A–C**), but for the CAK D97N-THZ1 cryo-EM reconstruction. (**J–L**) Additional views of the ligands in the cryo-EM density. Source data are available online for this figure.

© The Author(s)

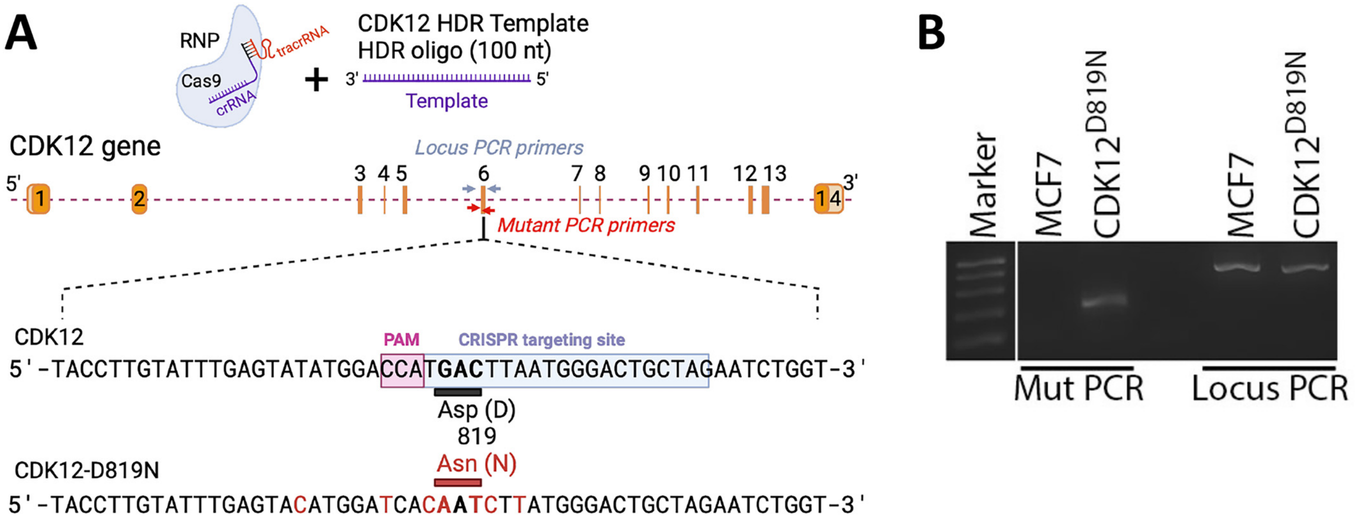

Figure EV4. CDK12-D819N CRISPR-Cas9 knock-in in MCF7 cells.

(**A**) Strategy for generating the D819N mutation in the CDK12 gene. The full sequence of the donor template is: 5′-TTCGTCTTTATGTAGGTGCCTTTTACCTTG-TATTTGAGTACATGGATCACAATCTTATGGGACTGCTAGAATCTGGTTTGGTGCACTTTTCTGAGGACCA-3′. (**B**) PCR was carried out using gDNA and primers amplifying the region around exon 6 ("locus PCR"; 5′-CGCCCAGCCACAGAAGATTA-3′ and 5′-GAGGAGAAGAGGAAAGTGCTTAA-3′, product size: 458 bp). PCR using primers, one of which is located within the region containing base changes incorporated in the donor template ("mutant PCR"; 5′-GGACTTGAGGCATTGTTATTT-3′ and 5′-CCATAAGATTGTGATCCATG-3′, product size: 116 bp), was carried out with gDNA prepared from cells following RNP CRISPR knock-in but prior to selection with (R)-CR8.

 © The Author(s)

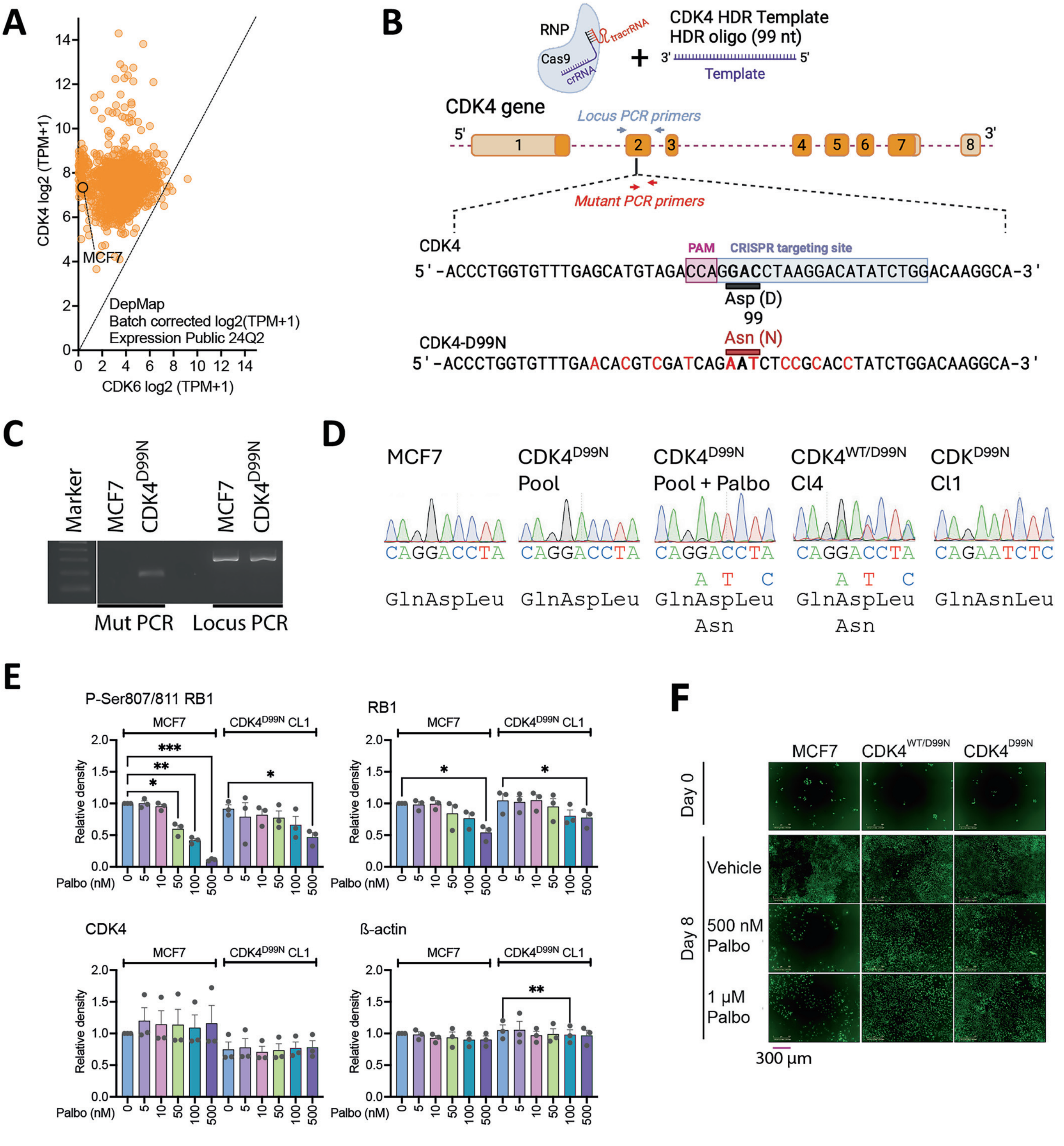
A
CDK4 log2 (TPM+1)
CDK6 log2 (TPM+1)
MCF7
DepMap
Batch corrected log2(TPM+1)
Expression Public 24Q2
B
RNP
Cas9
tracrRNA
crRNA
CDK4 HDR Template
HDR oligo (99 nt)
Template
CDK4 gene
Locus PCR primers
Mutant PCR primers
CDK4
PAM
CRISPR targeting site
5'-ACCCTGGTGTTTGAGCATGTAGACCAGGACCTAAGGACATATCTGGACAAGGCA-3'
Asp (D)
99
Asn (N)
CDK4-D99N
5'-ACCCTGGTGTTTGAACACGTCGATCAGAATCTCCGCACCTATCTGGACAAGGCA-3'
C
Marker
MCF7
CDK4D99N
Mut PCR
Locus PCR
D
MCF7
CDK4D99N Pool
CDK4D99N Pool + Palbo
CDK4WT/D99N Cl4
CDKD99N Cl1
CAGGACCTA
CAGAATCTC
GlnAspLeu
GlnAsnLeu
Asn
E
P-Ser807/811 RB1
RB1
CDK4
ß-actin
MCF7
CDK4D99N CL1
Relative density
Palbo (nM)
F
MCF7
CDK4WT/D99N
CDK4D99N
Day 0
Day 8
Vehicle
500 nM Palbo
1 μM Palbo
300 μm

Figure EV5. CDK4-D99N CRISPR-Cas9 knock-in in MCF7 cells.

(**A**) Batch corrected expression public 24Q2 data was acquired for CDK4 and CDK6 from the DepMap portal. (**B**) Strategy for generating the D99N mutation in the CDK4 gene. The full sequence of the donor template is: 5′-CCCGAACTGACCGGGAGATCAAGGTAACCCTGGTGTTTGAACACGTCGATCAGAATCTCCGCACCTATCTGGA-CAAGGCACCCCCACCAGGCTTGCCAGCCGAAA-3′. (**C**) Knock-ins were confirmed by PCR of gDNA using mutant-specific primers having the sequences, 5′-ACACGTCGATCAGAATCTCC-3′ and 5′-ATCCACCTCTCAATGCCTAC-3′. Locus-specific primers used for gDNA PCR had the sequences 5′-AGGTGGGGTGTGAT-GATCTG-3′, 5′AAGGGGAGGTACAGATGCAC-3′. (**D**) Sanger sequencing of DNA generated using locus PCR primers. Sequencing was performed using gDNA prepared from MCF7 cells after nucleofection (CDK4-D99N Pool), gDNA prepared from the CDK4-D99N Pool after culturing in the presence of 1 µM Palbociclib for 9 passages over a period of 3 months. Individual clones were isolated from the Palbociclib-selected pools; sequences of heterozygous and homozygous mutant clones are shown. (**E**) ImageJ was used to quantify the immunoblotting results for $n = 3$ independent experiments in which MCF7 cells and MCF7-CDK4-D99N CL1 cells were treated with Palbociclib for 48 h. Error bars = SEM. Pairwise comparisons between treated groups and vehicle were performed using repeated measures of one-way ANOVA followed by Fisher's LSD post hoc test (uncorrected). For P-Ser807/811 RB1 MCF7 cells: Veh vs Palbo (50 nM) $P = 0.0213$; Veh vs Palbo (100 nM) $P = 0.0026$; Veh vs Palbo (500 nM) $P = 0.0001$. CDK4^{D99N} CL1 cells: Veh vs Palbo (500 nM) $P = 0.0302$. For RB1, MCF7 cells: Veh vs Palbo (500 nM) $P = 0.0236$. Veh vs Palbo (500 nM) $P = 0.0143$. For β-actin, CDK4^{D99N} CL1 cells: Veh vs Palbo (100 nM) $P = 0.0063$. No asterisk indicates a non-significant difference ($P > 0.05$). (**F**) Representative images of cultures 24 h after seeding, when Palbociclib was added (day 0) and 8 days following the start of treatment. Source data are available online for this figure.

 © The Author(s)
